# Cholera in Holland

**Published:** 1846-12

**Authors:** 


*Cholera in Holland.*—This disease, under the mild form of what has been termed by the French *cholerine*, has made its appearance at Amsterdam and Rotterdam, and has already attacked a great number of individuals, but not one of the cases has proved fatal.—*Naval & Military Gaz.—Gaz. Méd.—Med. News.*

